# Procalcitonin as a Biomarker for a Bacterial Infection on Hospital Admission: A Critical Appraisal in a Cohort of Travellers with Fever after a Stay in (Sub)tropics

**DOI:** 10.1155/2009/137609

**Published:** 2009-11-18

**Authors:** Dennis A. Hesselink, Hanna Bosmans-Timmerarends, Jan-Steven Burgerhart, Pieter L. Petit, Perry J. van Genderen

**Affiliations:** ^1^Department of Internal Medicine, Harbour Hospital, Institute for Tropical Diseases, Haringvliet 2, 3011 TD Rotterdam, The Netherlands; ^2^Department of Internal Medicine, Erasmus Medical Centre, P.O. Box 2040, 3000 CA Rotterdam, The Netherlands; ^3^Department of Microbiology, Vlietland Hospital, P.O. Box 215, 3100 AE Schiedam, The Netherlands

## Abstract

Fever in a returned traveller may be the manifestation of a self-limiting, trivial infection but it can also presage an infection that can be rapidly progressive and lethal. We studied the diagnostic accuracy of procalcitonin (PCT) as a biomarker for a bacterial cause of fever in a cohort of 157 consecutive travellers with fever after a stay in the (sub)tropics. Elevated procalcitonin levels were observed not only in about 50% of travellers with proven bacterial infection, but also in a significant proportion of travellers with a likely infection. Using a cutoff point of 0.5 ng/mL, procalcitonin had a sensitivity of 0.52 and a specificity of 0.76 for a bacterial cause of fever on admission. Interestingly, only 1 out of 16 patients with a proven viral infection had a marginally elevated PCT concentration on admission, suggesting that an increased PCT level likely excludes a viral infection as the cause of fever. However, the diagnostic accuracy of this semiquantitative procalcitonin test for a bacterial cause of fever on admission is too poor to advocate its use in the initial clinical evaluation of fever in a setting of ill-returned travellers.

## 1. Background

While fever in a returned traveller may be the manifestation of a self-limiting, trivial infection, it can also presage an infection that can be rapidly progressive and lethal. Initial attention should focus most urgently on infections that are treatable and transmissible and that may cause serious sequelae or even death. International travel expands the list of potential infections that must be considered in returning travellers [1]. Malaria is probably the most feared infectious disease to consider in anyone who returns with fever after visiting an tropical area, necessitating investigation without delay [1]. After exclusion of malaria, the clinician must subsequently decide whether the imminent threat of the infection is likely to be caused by bacteria or viruses and whether the feverish patient should be admitted for intensified treatment including empirical treatment with antibiotics.

Procalcitonin (PCT), a precursor peptide from the hormone calcitonin, has been considered by some to be a specific and useful indicator of invasive infections by bacteria [2]. Under normal metabolic conditions, hormonally active calcitonin is produced and secreted in the C-cells of the thyroid gland through a regulated pathway after procession of the prohormone PCT [3]. Thus, under normal conditions, PCT levels in the circulation are very low (usually <0.05 ng/mL). Interestingly, in case of severe bacterial infections, microbial products (e.g., LPS) and proinflammatory mediators of the host response (e.g., Tumor Necrosis Factor *α* and Interleukin-1*β*) result in a generalized tissuewide induction of calcitonin mRNA and a consequent secretion of calcitonin precursors including unprocessed PCT through a nonregulated constitutive pathway [3]. As a result, PCT is released from all parenchymal tissues and differentiated cell types throughout the body, resulting in significant elevations of PCT levels in the circulation [3]. Moreover, Interferon-gamma inhibits IL-1*β*-induced calcitonin mRNA expression and PCT secretion, which may explain why circulating PCT levels tend to remain unchanged in viral infections [3].

In several clinical studies involving selected groups of patients with lower respiratory tract infections, pneumonia, acute exacerbations of chronic obstructive lung disease, and septic patients, respectively, antibiotic use was markedly diminished and prescribed shorter when treatment was guided by an ultrasensitive PCT assay [2, 4–6]. Apart from its surplus value in treatment algorithms, PCT may probably also contribute to an early diagnosis of bacterial infections in certain patient groups. We evaluated the diagnostic accuracy of PCT for bacterial infections in the initial clinical assessment of travellers, who returned home with fever after a stay in the (sub)tropics.

## 2. Methods

The Harbour Hospital is a 161-bed general hospital located in Rotterdam, The Netherlands. It also harbours the Institute of Tropical Diseases which serves as a national referral centre for tropical diseases. All travellers who presented with a documented fever on admission, were eligible for enrolment in this observational study. Fever was defined as a body temperature of ≥38.5°C. Since in our clinical practice malaria is ruled out first in patients who present with fever after a stay in the (sub)tropics, patients with malaria were excluded from this study. All laboratory measurements were performed using standard laboratory techniques. PCT was measured with the BRAHMS PCT-Q test (Brahms Diagnostics, Germany) according to the instructions of the manufacturer [7]. This immunochromatographic test allows a semiquantitative detection of PCT. It is designed as a point-of-care test for the rapid and easy measurement of PCT. Using this assay, an early laboratory-independent measurement of PCT on serum or plasma is possible within 30 minutes. PCT-Q results were classified as follows: a negative result or a PCT <0.5 ng/mL was classified as “*normal*”; a PCT of 0.5 ng/mL or a PCT between 0.5–2.0 ng/mL was classified as “*low*”; a PCT of 2.0 ng/mL or a PCT between 2.0–10.0 was classified as “*moderate*”; a PCT of 10.0 ng/mL and above 10 ng/mL was classified as “*high*” as recommended [8].

Demographic, clinical, and laboratory data on admission were collected for each traveller. The outcome of the patient was determined by two investigators (H.B.T and P.J.v.G) after careful review of patient records. Outcome was classified as follows: “*infection proven*” for those outcomes in which the causative micro-organism was either cultured or a seroconversion was documented or in case of a pathognomonical clinical presentation (e.g., a *creeping eruption*); outcomes in which a specific micro-organism could not be demonstrated, but the clinical course suggested an infectious cause were classified as “*infection likely.*” These presumptive diagnoses were based on epidemiologic and clinical features, supporting laboratory investigations, and a response to appropriate treatment. In case no infectious cause could be found (usually after extensive investigations) or an alternative diagnosis was made, the outcomes were classified as “*no infection.*” In those travellers in which the outcome was classified as “*infection proven”* subgroups were made depending on the class of the causative micro-organisms, that is, viral, bacterial, or parasitic. Finally, of each traveller, the PCT level on admission was related to these predefined outcomes, and sensitivity analyses of PCT for bacterial infections were performed using different cutoff levels of PCT.

### 2.1. Statistical Analysis

All values are presented as median (range). For comparison between groups, the Kruskal-Wallis test was used as appropriate. Post hoc analysis was performed using Mann-Whitney *U* test. *P* values at *α* < .05 were considered statistically significant.

## 3. Results

**Figure 1:**
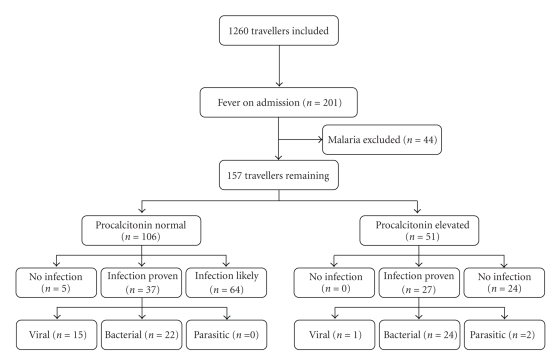
Flow-chart of procalcitonin test results on admission in relation to a predefined outcome in a cohort of 157 consecutive travellers who presented with fever (i.e., a body temperature ≥38.5°C on admission). An elevated procalcitonin level was defined as a PCT level ≥0.5 ng/mL as measured with a semiquantitative assay.

In the period January 2005 to December 2007 a total of 1260 travellers were screened for inclusion in this observational study, as shown in Figure 1. In 201 of them fever was documented on admission. Forty-four of these patients had malaria and were excluded from this study according to our clinical algorithm. When the 157 remaining patients were allocated to the predefined outcomes, an infectious cause was demonstrated in 64 patients (group “infection proven”), an infectious cause was considered likely in 88 patients (group “infection likely”), and a cause for fever other than an infection was demonstrated in 5 patients (group “no infection”), respectively. The general characteristics of the various patient groups on admission, grouped according to their (predefined) outcomes, are shown in Table 1.

### 3.1. Patients Grouped as “Infection Proven”

Within the group “infection proven” a viral cause was demonstrated in 16 (*dengue virus n* = 8; *Epstein Barr Virus n* = 2; *influenza n* = 2; *rubella n* = 1; other *n* = 3) travellers, 46 patients had a bacterial cause (*Salmonella n* = 13; *Streptococcal* infection *n* = 6; *Rickettsia n* = 4; *Campylobacter n* = 4; *Shigella n* = 3; *Staphylococcal* infection *n* = 2; *Leptospira n* = 4; *Coxiella burneti n* = 3; other *n* = 7), and parasitic cause was demonstrated in 2 cases (Katayama syndrome due to acute schistosomiasis in 1; visceral leishmaniasis in 1), respectively.

### 3.2. Patients Grouped as “Infection Likely”

In 88 patients grouped as “infection likely” the clinical presentation and subsequent course was highly suggestive of an infectious cause but a causative micro-organism could not be demonstrated (gastroenteritis *n* = 26; upper and lower respiratory tract infections *n* = 21; unspecified febrile illness *n* = 17; skin infection *n* = 4; urinary tract infections *n* = 4, other *n* = 16), even after repeat cultures and serology.

### 3.3. Patients Grouped as “No Infection”

The group “no infection” consisted of 5 patients in whom fever was related to a noninfectious cause (inflammatory bowel disease *n* = 2; auto immune disorder *n* = 3).

### 3.4. PCT Results on Admission

**Figure 2:**
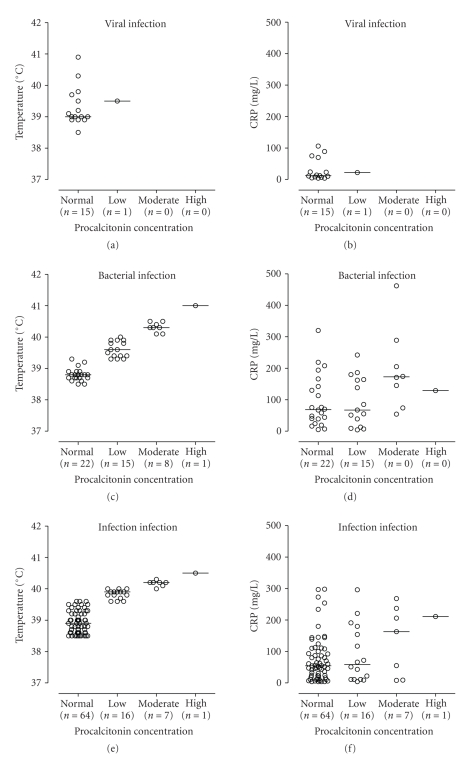
*Left panel.* Relationship between body temperature on admission and procalcitonin levels on admission. (a) Relationship in patients with a documented viral cause of fever (*n* = 16). No statistical analysis performed. (c) Relationship in patients with a documented bacterial cause of fever (*n* = 46). Overall, *P*-value <.0001 (Kruskal Wallis), specified per PCT result: Normal versus Low *P* < .0001 (Mann-Whitney U test). Normal versus Moderate *P* < 0.0001 (Mann-Whitney U test), and Low versus Moderate *P* = .0001 (Mann-Whitney U test), (e) Relationship in patients with a likely infection (*n* = 88). Overall, *P*-value <.0001 (Kruskal Wallis), specified per PCT result: Normal versus Low *P* < .0001 (Mann-Whitney U test), Normal versus Moderate *P* < .0001 (Mann-Whitney U test), Low versus Moderate *P* = .0003 (Mann-Whitney U test). *Right panel.* Relationship between C-reactive protein and procalcitonin levels on admission. (b) Relationship in patients with a documented viral cause of fever (*n* = 16). No statistical analysis performed. (d) Relationship in patients with a documented bacterial cause of fever (*n* = 46). Overall, *P*-value *P* = .12 (Kruskal-Wallis). (f) Relationship in patients with a likely infection (*n* = 88). Overall, *P*-value .36 (Kruskal-Wallis).

The majority (67.5%) of the 157 patients with fever had a normal PCT level on admission. Patients with fever caused by a viral infection or with a noninfectious cause of fever had a normal PCT on admission in all but 1 patient. Moderate to high PCT levels were only observed in patients with documented bacterial and parasitic infections and in about a quarter of the patients grouped as “likely infection.” As shown in Figure 2 (left panel), in patients with proven bacterial and viral cause of fever and those with a likely infection, a significant relation could be established between body temperature on admission and PCT levels, respectively. This correlation was most clear for patients with a demonstrated bacterial cause of fever. This relationship was not present for CRP and PCT levels on admission, respectively (Figure 2 right panel). 

### 3.5. Diagnostic Accuracy of PCT for a Bacterial Cause of Fever on Admission

**Figure 3:**
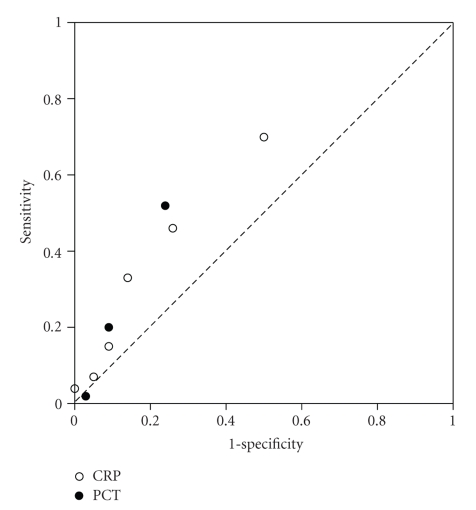
Receiver operating characteristic (ROC) curves of procalcitonin (PCT) and C-reactive protein (CRP) testing on admission for a confirmed bacterial cause of fever in 157 travellers who returned home with fever after a stay in the (sub)tropics. The respective cutoff points for CRP were 300, 250, 150, 100, and 50 mg/L, respectively, and the cutoff points for PCT were 10, 2, and 0.5 ng/mL, respectively.

As detailed in Table 2(a), a PCT cutoff point of 0.5 ng/mL resulted in a limited sensitivity and specificity of 0.52 and 0.76, respectively. The positive and negative predictive values of PCT for a bacterial cause of fever were too low, with values of 0.47 and 0.79, respectively. Increasing the cutoff level to 2.0 and 10.0 ng/mL, respectively, increased the specificity but resulted in a dramatic drop of sensitivity. The PPV of PCT (cutoff point 0.5 ng/mL) for a confirmed bacterial cause of fever would have increased to 0.94 if all patients with a likely infection were assumed to have proven bacterial infection (Table 2(b)). In a receiver operating characteristic (ROC) curve, PCT test appeared only somewhat more sensitive for predicting a bacterial cause of fever on admission than testing of C-reactive protein (Figure 3).

### 3.6. Diagnostic Accuracy of PCT for a Positive Blood Culture (Bacteremia) on Admission

In the group of patients with a documented bacterial infection, blood cultures were performed in 41 of 46 patients on admission. Increased PCT levels on admission were significantly (Fisher exact test, *P* = .031) more frequently observed in bacteremic (i.e., a positive blood culture) individuals (11 of 17 patients) than in individuals with a negative blood culture on admission (7 of 24 patients). As shown in Table 3, an increased PCT level on admission had a sensitivity of 0.65 and PPV of 0.61 for bacteremia at a cutoff point of 0.5 ng/mL. Increasing the cutoff point to 2 and 10 ng/mL dramatically affected the diagnostic performance of PCT test. For the whole cohort of 157 travellers with fever—assuming that all other patients had a negative blood culture on admission—an increased PCT had a sensitivity of 0.65 and PPV of 0.22 for bacteremia, whereas specificity and NPV were 0.71 and 0.94, respectively (data not shown). 

## 4. Discussion/Conclusion

PCT has been used for more than a decade for the diagnosis, monitoring of the course and severity of the systemic inflammatory response syndrome (SIRS) observed during severe infections [2–6]. We were particularly interested in the accuracy of PCT for predicting a bacterial cause of fever in a cohort of consecutive travellers who returned home with fever. Although PCT levels above 2.0 ng/mL (corresponding with the class “moderate” or “high”) were only seen in patients with documented bacterial and parasitic causative micro-organisms and in some patients allocated to the group “likely infection”, respectively, the diagnostic accuracy of PCT for bacterial infections was too poor to advocate this semiquantitative PCT test for use in the initial clinical evaluation of fever. At each cutoff point of PCT, in particular sensitivity and PPV were disappointingly low. This assumption became also evident in the poor performance of PCT in the ROC characteristics, where the performance of PCT was not superior over measurement of C-reactive protein. The conclusion that the diagnostic accuracy of PCT for bacterial cause of fever is too low is also in line with the observation made by others in other settings, such as for the diagnosis of bacteremia in an emergency department population [9].

The overall sensitivity of PCT for a bacterial cause of fever could probably be improved if PCT was measured more precisely with an ultrasensitive PCT assay instead of the semiquantitative assay used in this study. Although a direct comparative study with a quantitative luminometric assay demonstrated that, given a cutoff value of 2.0 ng/mL, the majority of the semiquantitative PCT results were correctly categorized [8], the ultrasensitive assays have the advantage that they may accurately detect PCT concentrations of 0.1 ng/mL and are capable of even measuring differences in PCT levels in the range below 0.5 ng/mL, the normal range in our study [2, 4–6]. By using these ultrasensitive assays, more subtle elevations of circulating PCT might have been demonstrable, as may occur in more localized bacterial infections. However, on the other hand, these sensitive assays may have the relative disadvantage that they are probably more time-consuming and laboratory-dependent. In contrast, the semiquantitative PCT assay may be performed at bedside or in the emergency room without a need for further technical equipment or support and may therefore be incorporated more easily in the initial clinical assessment of a traveller with fever.

The poor diagnostic accuracy of PCT for identifying bacterial causes of fever in our study may have resulted from a relatively large group of patients with bacterial infections of only limited severity, causing a local but not a systemic inflammatory response. This suggestion is supported by the finding that in patients who had an elevated PCT on admission, a higher body temperature—one of the key contributors to SIRS—was documented, as compared with patients with normal PCT levels. In fact, a significant relationship could be established between body temperature on admission and PCT level, in particular in patients with a documented bacterial cause of fever. In line with this, in the 41 patients with a documented bacterial cause of fever whom blood cultures were collected on admission, increased PCT levels on admission were significantly more frequently seen in bacteremic patients than in nonbacteremic individuals, confirming that systemic infections are indeed a more powerful stimulus for PCT release than localized infections.

Another limitation of our study may be the modest prevalence (29.3%) of proven bacterial causes in our study, which, of course, directly influences the positive and negative predictive values of PCT as a biomarker for bacterial cause of fever. With the use of more sophisticated diagnostic techniques like PCR, a causative bacterial micro-organism might have been demonstrated in a subset of travellers now allocated to the group “infection likely.” This would have significant impact on the discriminative power of PCT by increasing the prevalence of proven bacterial infections in our study. For example, the positive predictive value of PCT (cutoff point 0.5 ng/mL) for a bacterial cause of fever would have increased to 0.94 if all patients with a likely infection were assumed to have proven bacterial infection (Table 2(b)).

PCT appeared to be a biomarker with a rather high specificity for bacterial infections as PCT levels were normal in all but one patient with a documented viral cause of fever. This observation is consistent with the findings of Linscheid et al. [3] and may be explained by the fact that interferon-gamma inhibits IL-1*β*-induced calcitonin mRNA expression and PCT secretion [3]. Although speculative, our data suggest that a PCT cutoff point of 2.0 ng/mL on admission may safely exclude viral infections and ailments due to noninfectious causes as a cause of fever in the ill-returned traveller. 

In conclusion, although an elevated PCT concentration likely excludes viral causes of fever, the diagnostic accuracy of this semiquantitative PCT test for a bacterial cause of fever was too poor to advocate the use of this test in the initial clinical evaluation of fever in a setting of travellers who return home with fever after a stay in the (sub)tropics.

## Figures and Tables

**Table 1:** General and laboratory characteristics of the travellers with fever on admission, specified by outcome and class of causative micro-organism. Data are given as median [range].

	Infection proven (*n* = 64)			Infection likely (*n* = 88)	No infection (*n* = 5)
	Viral infection (*n* = 16)	Bacterial infection (*n* = 46)	Parasitic infection (*n* = 2)		
Age (year)	27 [17–50]	30 [15–68]	30 [17–43]	40 [14–73]	40 [10–53]
Male/female	8/8	24/22	1/1	57/31	1/4

Continent of acquisition (*n* [%])					

Europe	1 [6.3]	1 [2.2]	0	4 [4.5]	0
Americas	6 [37.5]	9 [19.6]	0	10 [11.4]	1 [20.0]
Africa	2 [12.5]	10 [21.7]	1 [50.0]	31 [35.2]	4 [80.0]
Asia and Oceania	7 [43.8]	26 [56.5]	0	43 [48.9]	0
Unknown	0	0	1 [50.0]	0	0

Presenting symptoms (*n* [%])					

History of fever	13 [81.37]	45 [97.8]	2 [100]	84 [95.5]	4 [80.0]
Skin problems	5 [31.3 ]	10 [21.7]	0	12 [13.6]	0
Diarrhea	7 [43.8]	25 [54.3]	2 [100]	35 [39.8]	2 [40.0]
Shortness of breath	5 [31.3]	14 [30.4]	1 [50.0]	31 [35.2]	2 [40.0]
Headache	10 [62.5]	26 [56.5]	1 [50.0]	50 [56.8]	3 [60.0]
Muscle ache	6 [37.5]	18 [39.1]	1 [50.0]	35 [39.8]	1 [20.0]
Malaise	4 [25.0]	9 [19.6]	0	32 [36.4]	2 [40.0]
Nausea/vomiting	6 [37.5]	16 [34.8]	1 [50.0]	25 [28.4]	0
Others	8 [50.0]	7 [15.2]	0	22 [25.0]	2 [40.0]

Duration of complaints (*n* [%])					

<8 days	13 [81.3 ]	34 [73.9]	2 [100]	71 [80.7]	2 [40.0]
8–14 days	2 [12.5]	7 [15.2]	0	9 [10.2]	1 [20.0]
15–28 days	0	2 [12.5]	0	1 [1.1]	1 [20.0]
>29 days	1 [6.3]	3 [18.8]	0	7 [8.0]	1 [20.0]

Physical findings (median [range])					

Temperature (°C)	39.1 [38.5–40.9]	39.3 [38.5–41.0]	39.2 [38.6–39.8]	39.2 [38.5–40.5]	38.7 [38.5–39.9]
Pulse (beats/minute)	100 [72–120]	104 [72–125]	122 [100–144]	93 [65–160]	94 [84–104]
Systolic blood pressure (mm Hg)	120 [95–160]	120 [90–180]	133 [130–136]	126 [90–195]	115 [96–130]

Laboratory findings					

ESR (mm/h)	10 [2–56]	23 [3–114]	38	21 [2–98]	28 [8–58]
Leukocytes (×10^9^/L)	6.0 [1.9–14.2]	9.8 [3.3–18.9]	7.7 [4.4–11.0]	8.0 [2.6–23.2]	5.8 [3.3–7.4]
C-reactive protein (mg/mL)	13 [4–106]	76 [4–462]	102 [100–104]	56 [4–298]	9 [4–125]

**Table:** (a) Descriptive statistics of procalcitonin (PCT) as an admission biomarker for a confirmed bacterial cause of fever in all 157 travellers. Data are given as proportion [95% confidence interval].

PCT test	Cutoff 0.5 ng /mL	Cutoff 2.0 ng/mL	Cutoff 10 ng/mL
Sensitivity	0.52 [0.37–0.67]	0.20 [0.10–0.34]	0.02 [0.00–0.13]
Specificity	0.76 [0.66–0.83]	0.91 [0.84–0.95]	0.97 [0.92–0.99]
Positive predictive value	0.47 [0.33–0.61]	0.47 [0.25–0.71]	0.25 [0.01–0.78]
Negative predictive value	0.79 [0.70–0.86]	0.73 [0.65–0.80]	0.71 [0.63–0.78]

**Table:** (b) Descriptive statistics of procalcitonin (PCT) as an admission biomarker for a confirmed bacterial cause of fever in all 157 travellers if all patients with a likely infection (*n* = 88) were assumed to also have a confirmed bacterial infection. Data are given as proportion [95% confidence interval].

PCT test	Cutoff 0.5 ng /mL	Cutoff 2.0 ng /mL	Cutoff 10 ng /mL
Sensitivity	0.36 [0.28–0.45]	0.13 [0.08–0.20]	0.01 [0.00–0.06]
Specificity	0.87 [0.65–0.97]	0.91 [0.70–0.98]	0.91 [0.70–0.98]
Positive predictive value	0.94 [0.83–0.98]	0.89 [0.65–0.98]	0.50 [0.09–0.91]
Negative predictive value	0.19 [0.12–0.28]	0.15 [0.10–0.23]	0.14 [0.09–0.20]

**Table 3:** Descriptive statistics of procalcitonin (PCT) as an admission biomarker for bacteremia in 41 travellers who had a confirmed bacterial cause of fever and a blood-culture done on admission. Data are given as proportion [95% confidence interval].

PCT test	Cutoff 0.5 ng /mL	Cutoff 2.0 ng /mL	Cutoff 10 ng /mL
Sensitivity	0.65 [0.39–0.85]	0.18 [0.05–0.44]	0.06 [0.00–0.31]
Specificity	0.71 [0.49–0.87]	0.92 [0.72–0.99]	1.00 [0.83 −1.00]
Positive predictive value	0.61 [0.36–0.82]	0.60 [0.17–0.93]	1.00 [0.05 −1.00]
Negative predictive value	0.74 [0.51–0.89]	0.61 [0.44–0.76]	0.60 [0.43–0.75]
